# Turnover Intention and Its Related Factors of Employed Doctors in Korea

**DOI:** 10.3390/ijerph16142509

**Published:** 2019-07-14

**Authors:** Suhyun Oh, Hyeongsu Kim

**Affiliations:** 1 Research Institute for Healthcare Policy, Korean Medical Association, Seoul 04373, Korea; 2 Department of Preventive Medicine, School of Medicine, Konkuk University, Seoul 05029, Korea

**Keywords:** employed doctor, turnover intentions, Korean Physician Survey

## Abstract

Employment turnover among doctors at healthcare facilities negatively influences healthcare provision, facility management, and staffing. To support institutional and policy change, turnover intentions and its related factors of employed doctors were evaluated with 2016 Korean Physician Survey (*n* = 2719) in Korea. About 30.5% intended a turnover within two years. The significant related factors by multivariate analysis via binary logistic regression were gender, age, specialty, type of facility, length of current employment, usual number of hours worked per week, and income satisfaction. The odds of reporting turnover intention are 46.2% greater for males than females and 55.5% greater for aged 30–39 than aged 40–49. The odds are 28.9% smaller for support medicine than internal medicine. The odds are 224.2% greater for those employed at tertiary hospitals than those employed at clinics, but the odds are 34.0% smaller for convalescent hospital employment than general hospital employment. The number of years of current employment and income satisfaction each negatively, and the number of hours worked per week positively, related to turnover intentions. Fair compensation and performance evaluation systems and reasonable working hours should be guaranteed at healthcare facilities to reduce turnover, and institutional and policy measures should be implemented to improve workplace environmental quality.

## 1. Introduction

Since 2012, the numbers of doctors employed at healthcare facilities have exceeded the numbers of self-employed doctors in Korea. Of the 105,583 members who registered at the Korean Medical Association (KMA), about 40.7% were employed and about 34.4% were self-employed in 2014 [1]. The turnover of employed doctors can significantly influence the quality of healthcare and facility management. That is, turnover of doctors can lead to unmet expectations for the patients caused by the inexperienced new doctors, which results in patient dissatisfaction [2]. Healthcare organizations can experience managerial and financial problems from unpredictable turnover consequences [3].

Although the relationships of predisposing factors to turnover intentions are determined after a turnover occurs to reveal the factors that influenced the intentions, the best way to determine these relationships is not straightforward. Turnover intention has been a direct predictor of the turnover in former studies and can be used as a proxy variable for turnover [4,5,6]. Turnover intention is defined as an individual’s intention to voluntarily terminate his or her employment relationship [7], which is more meaningful when it is understood as more than the turnover decision or event because it somewhat reflects the nature of the employers’ organizational management [8,9]. Moreover, because turnover intentions relate to turnover behaviors, understanding turnover intentions among current employees might help to identify ways to decrease turnover intentions and, thus, actual turnovers [10].

The focus of previous studies outside Korea on health workers’ turnover intention and decision to stay or leave their posts included what factors affected their decision from the viewpoint of individual, organization, and job [8,11,12,13,14,15]. Job satisfaction and its particular dimensions, such as work nature, autonomy, supervision, remunerations, work environment, peer group relationships, organizational commitment, as well as its individual components (affective commitment, continuance commitment, and normative commitment) and various demographic and personal characteristics were determinants of turnover intention.

There are only three previous studies on Korean doctors’ turnover intentions [10,16,17]. One study dealt with turnover intentions of doctors at general hospitals after the separation of prescribing and dispensing drugs [10]. The other two studies targeted turnover intentions of doctors in public hospitals [16] and in the public health center [17]. So far, there is no approach to turnover intentions of doctor at all types of healthcare facilities.

This study investigated the turnover intentions of employed doctors in Korea to make an evidence for policies to prevent and control their turnover intentions.

## 2. Materials and Methods

### 2.1. Study Design, Data, Study Population

This study used a cross-sectional the 2016 KPS data. The 2016 KPS was a web-based self-administered questionnaire survey by the Research Institute for Healthcare Policy (RIHP) of Korean Medical Association (KMA) from 21 November 2016 to 8 January 2017. This was the first national survey for all medical doctors in Korea. Of 108,870 medical doctors who registered their information in database in KMA, 30,873 that did not agree to disclose their personal information or email address were excluded. The email was sent to 77,997 members and 61,983 checked the email. Though the stratified quota sampling (sex, age, employment status), 8564 members (13.8%) participated in 2016 KPS [18]. Of them, 2920 respondents were employed doctors. Of the employed doctors, 201 were not currently at a healthcare-related job, were working outside Korea, had turnover intentions based on foreign training or community service, or were aged 65 years or older.

### 2.2. Variables

#### 2.2.1. Dependent Variable

Turnover intention was defined as “the intention to leave paid employment as a physician for different employment, such as private medical practice, a teaching post as a professor, and so on.” The respondents were asked: “What is your turnover plan within the next two years?” The six response options were: 1 = *I plan to start my own practice*, 2 = *I plan to be a professor*, 3 = *I plan to take a research job*, 4 = *I plan to take an administrative job*, 5 = *I plan to be a journalist*, 6 = *I plan to retire*, and 7 = *I do not plan to change jobs within the next two years*. For the analysis, responses 1 through 6 were categorized as “yes” and those that chose response 7 were categorized as “no.”

#### 2.2.2. Independent Variables

Through literature review, we gathered some factors related with turnover intention of doctors [8,11,12,13,14,15] and we selected some of them from 2016 KPS. The independent variables included the demographic characteristics of gender and age. The respondents’ medical specialties were classified as internal medicine, surgery, support medicine, and no specialty certification. Internal medicine included internal medicine, neurology, psychiatry, pediatrics, dermatology, tubercular medicine, rehabilitation, and family medicine. Surgery included general, orthopedic, thoracic, plastic, obstetric and gynecological, ophthalmological, otorhinolaryngological, urological, neurosurgery, and emergency medicine. Support medicine included anesthesiology and pain management, radiology, radiation oncology, pathology, laboratory medicine, preventive medicine, nuclear medicine, and occupational and environmental medicine. The type of healthcare facility, nature of healthcare facility, region, length of current employment, usual hours worked per week, and satisfaction with income were tested. Types of healthcare facilities were private clinics, hospitals, general hospitals, tertiary hospitals, and convalescent hospitals. The nature of the facility was either public or private. Regions were the *dong* or the *eup/myeon*. Response options to the question about satisfaction with income were on a five-point scale ranging from very dissatisfied to very satisfied. For the analysis, responses of “very dissatisfied” and “dissatisfied” were combined into “dissatisfied” and responses of “satisfied” and “very satisfied” were merged into “satisfied” to create a three-category variable (dissatisfied, neutral, satisfied).

### 2.3. Method of Statistical Analysis

Descriptive statistics were generated on all of the variables and bivariate Chi-square for contingency tests were performed to determine the factors that significantly influenced turnover intentions. Then, a multivariate analysis via binary logistic regression was performed to estimate the likelihoods of the independent variables’ association on turnover intentions with respect to the independent variables whose Chi-square test results had *p*-values smaller than 0.20, gender, and age. The odds ratios (OR) and the 95% confidence intervals were generated. All analyses were computed using IBM SPSS version 19.0 (IBM Corporation, Armonk, NY, USA) and statistical significance was determined at *p* < 0.05.

### 2.4. Research Ethics

The Institutional Review Board of Konkuk University exempted this study (No. 7001355-201804-E-075) because it was limited to partial analysis of processed secondary data of the 2016 KPS with no personal identifiers or sensitive information.

## 3. Results

### 3.1. Turnover Intentions

Table 1 shows the results of the Chi-square test analyzing the association between independent variables and dependent variables. The percentage of the sample with turnover intentions within the next two years was 30.5% (Table 1). Males were more likely than females to intend a turnover (31.1% versus 28.1%), but the difference was not statistically significant (*p* = 0.175). However, there was a significant difference by age group (*p* < 0.000), which was highest among those younger than 50 (42.2%) and lowest for those aged 50–59 (14.0%). Regarding differences by specialty, the respondents in support medicine were least likely to report turnover intentions (23.6%), which was statistically significant (*p* = 0.004). Turnover intentions were highest among those employed at tertiary hospitals (59.9%), followed by clinics (39.6%) and hospitals (26.7%) (*p* < 0.000). The likelihood of intending a turnover decreased as length of current employment increased (*p* < 0.000), and turnover intentions were highest among those with five or less years of employment at the current job (33.8%). Turnover intentions increased with the number of work hours (*p* < 0.000); respondents working 65 or more hours per week were the most likely to intend a turnover (48.4%). The relationship of income satisfaction to turnover intentions was similar; intentions increased as satisfaction decreased (*p* < 0.000). About 41.0% of the dissatisfied respondents had turnover intentions, followed by those who were neutral (27.4%) and those who were satisfied (21.5%). There was no statistically significant difference in turnover intentions regarding region or private versus public institution.

### 3.2. Factors Associated with the Turnover Intentions of Employed Doctors

The multivariate analysis via binary logistic regression multinomial logistic regression analysis found that gender, age, specialty, type of healthcare facility, length of current employment, usual number of hours worked per week, and income satisfaction were significantly related to turnover intentions (Table 2). The odds of reporting turnover intention are 46.2% greater for males than females. The odds are 55.5% greater for aged 30–39 than aged 40–49. The odds are 28.9% smaller for support medicine than internal medicine. The odds are 224.2% greater for those employed at tertiary hospitals than those employed at clinics, but the odds are 34.0% smaller for convalescent hospital employment than general hospital employment. The odds are 64.1% smaller for those working with 16 or more years than those working with six to 10 years. The odds are 30.2% smaller for those working less than 40 h per week than those working 41–52 h per week and 43.1% for those working more than 65 h per week than those working 41–52 h per week. The odds are 100.9% greater for those unsatisfied with their incomes than those satisfied with their incomes.

## 4. Discussion

This study aimed to reveal the turnover intentions and related factors in a sample of employed doctors in Korea to make an evidence for prevention and control measures. First, about 30.5% of the respondents reported intentions to leave their current employment within the next two years to open a private practice, take a teaching position, or engage in other work. Although the target and method of survey were different, a previous relevant study conducted by questionnaire survey in 2011 found the turnover rate among 333 doctors at 31 national hospitals in Korea was 20.8% [16] and a national survey of 1174 family physicians working in the National Health Service of England in 2001 (for within five years) was 16.5% [8]. The current study might have found a high percentage of turnover intentions because of low-quality work conditions and environments to maintain their position, thus deteriorating their job security.

The significant factors related with turnover intentions were gender, age, specialty, type of healthcare facility, length of current employment, usual number of hours worked per week, and income satisfaction in this study. Males were more likely than females to intend a turnover, which might support a previous study that found female doctors tended to have low expectations regarding work conditions because they had few opportunities to obtain jobs with conditions better than male doctors and had relatively high job satisfaction [19]. It is important to develop organizational cultures and work environments that accommodate the particular needs of male doctors.

Regarding age differences in turnover intentions, the youngest respondents were more likely than those aged 40 to 49 to intend to make a change, which supports previous studies’ results [10,17]. Older employees tend to highly value stability, and they have relatively more opportunities for promotions and high incomes, which might relate to their lower turnover intentions. It seems important to focus on retaining the younger doctors, which might be accomplished by changing the compensation and performance evaluation system.

Turnover intentions varied by type of facility. Respondents at clinics and those at tertiary hospitals had greater turnover intentions than those at general hospitals and doctors at convalescent hospital had lower turnover intention. These results that employed doctors apparently preferred general hospitals to clinics and tertiary hospitals conflicts with previous evidence that most employed doctors prefer large tertiary hospitals. Despite the relatively high job security of large facilities compared to small and mid-sized hospitals, it seems likely that a high rate of turnover intentions stems from the greater demands placed on doctors at tertiary hospitals in Korea, which have more patients with more varied problems. In addition, the relatively low work intensity and environment of convalescent hospitals, which mainly treat patients with chronic diseases, seem to have a lower turnover intention compared to general hospitals that treat patients with diverse and complex problems. However, additional studies are needed to draw conclusions.

The turnover intention of employed doctors was proportional to the working hours per week. Healthcare professionals often need to work long hours compared to other professionals because of the small hospital staffs hired to compensate for the high costs of labor to total expenditures. On 1 July 2018, the Labor Standards Act was revised to mandate enterprises and institutions with more than 300 employees to limit the number of hours worked per week to 52. However, it exempted the healthcare sector from that requirement. Long workweeks might degrade job performance, increase the likelihood of medical malpractice, and negatively influence workers’ health. A crisis in providing high-quality and safe healthcare and in excessive turnovers might result [20]. Institutional and policy measures should be established to prevent excessive turnover of employed doctors by adopting the Labor Standards Act’s limits and improving workplace conditions.

Income satisfaction negatively related to turnover intentions, which supported similar findings that satisfaction with income was an important factor to turnover intentions in the healthcare sector [11,21]. It is important for organizations to appropriately pay their workers. Because doctors are professionals with significant expertise and skills, their compensation should be based on their skills levels, workloads, and work intensities. It is necessary to have performance review/compensation systems, welfare systems, and training opportunities that reflect doctors’ value to society.

This study has some limitations. First, the cross-sectional design employed secondary data drawn from the KPS and it was unable to analyze various factors that might influence turnover intentions. Future studies would make progress toward a full understanding of the situation by including a wider range of factors. Second, this study did not specifically examine the high rate of turnover intentions at tertiary hospitals, and targeted research is needed on the topic.

## 5. Conclusions

This study found that about 30.5% of the sample reported turnover intentions within the next two years and that gender, age, specialty, type of facility, length of current employment, usual number of hours worked per week, and satisfaction with income were related to those intentions. Although malleable factors to control the turnover intention were restricted to working hour and satisfaction with income, our results could serve as a reference for institutional and policy measures aimed at preventing or controlling doctors’ turnover intentions. Diverse studies are needed to reveal the factors to manage the turnover intention.

Because about 40.7% of Korea’s doctors are employed, their stable employment is important to public health. Turnover has some negative consequences, which include weakening organizational competitiveness and management and disruption of the supply and demand of human resources at the national level. Therefore, strategic responses are needed to predict and control turnover at hospitals and to guide human resources management. This study is the first to identify factors that are associated with employed doctors’ turnover intentions in Korea using 2016 KPS data. A reasonable performance evaluation and compensation system and less burdensome work hours should be implemented at healthcare facilities to increase job satisfaction and lower turnover intentions among employed doctors. Institutional and policy changes should be made to improve unreasonable workplace conditions.

## Figures and Tables

**Table 1:** Variation in turnover intentions in the analyzed variables (*n* = 2719).

Variables		Turnover Intentions				Total	%	*p*-Value *
		Yes		No				
		*N*	%	*N*	%			
Total		828	30.5	1891	69.5	2719		
Gender	Male	659	31.1	1459	68.9	2118	77.9	0.175
	Female	169	28.1	432	71.9	601	22.1	
Age	≤39	534	42.2	731	57.8	1265	46.5	0.000 *
	40–49	221	25.0	664	75.0	885	32.5	
	50–59	52	14.0	320	86.0	372	13.7	
	≥60	21	10.7	176	89.3	197	7.2	
Specialty	Internal	397	32.7	816	67.3	1213	44.6	0.004 *
	Surgery	309	31.0	689	69.0	998	36.7	
	Support	103	23.6	334	76.4	437	16.1	
	No specialty certification	19	26.8	52	73.2	71	2.6	
Type of healthcare facility	Clinic	194	39.6	296	60.4	490	18.0	0.000 *
	Hospital	154	26.7	422	73.3	576	21.2	
	General hospital	251	23.7	808	76.3	1059	38.9	
	Tertiary hospital	188	59.9	126	40.1	314	11.5	
	Convalescent	41	14.6	239	85.4	280	10.3	
Nature of healthcare facility	Public	109	32.2	230	67.8	339	12.5	0.488
	Private	719	30.2	1661	69.8	2380	87.5	
Region	*Dong*	758	30.5	1726	69.5	2484	91.4	0.882
	*Eup/myeon*	70	29.8	165	70.2	235	8.6	
Length of current employment (years)	1–5	695	33.8	1362	66.2	2057	75.7	0.000 *
	6–10	96	23.9	306	76.1	402	14.8	
	11–15	30	19.4	125	80.6	155	5.7	
	≥16	7	6.7	98	93.3	105	3.9	
Usual number of hours worked per week	≤40	115	20.9	434	79.1	549	20.2	0.000 *
	41–52	501	29.7	1186	70.3	1687	62.0	
	53–64	122	41.1	175	58.9	297	10.9	
	≥ 65	90	48.4	96	51.6	186	6.8	
Income satisfaction	Unsatisfactory	361	41.0	520	59.0	881	32.4	0.000 *
	Neutral	334	27.4	885	72.6	1219	44.8	
	Satisfactory	133	21.5	486	78.5	619	22.8	

* Chi-square test was applied.

**Table 2:** Relationships of demographic characteristics and work-related variables to turnover intentions; odds ratios and confidence intervals (*n* = 2719).

Variables		Turnover Intentions			
		Odds Ratio	95% Confidence Interval		*p*-Value *
			Lower	Upper	
Gender	Male	1.462	1.167	1.831	0.001
	Female (ref.)	1.000			
Age					
	≤39	1.555	1.254	1.929	0.000
	40–49 (ref.)	1.000			
	50–59	0.581	0.409	0.826	0.003
	≥60	0.439	0.266	0.725	0.001
Specialty	Internal (ref.)	1.000			
	Surgery	0.816	0.667	0.998	0.048
	Support	0.711	0.542	0.934	0.014
	No specialty certification	0.748	0.416	1.347	0.334
Type of healthcare facility	Clinic	2.083	1.627	2.666	0.000
	Hospital	1.172	0.918	1.495	0.203
	General hospital (ref.)	1.000			
	Tertiary hospital	3.242	2.413	4.356	0.000
	Convalescent hospital	0.660	0.447	0.974	0.036
Length of employment (years)	1–5	1.269	0.961	1.674	0.093
	6–10 (ref.)	1.000			
	11–15	0.867	0.534	1.407	0.564
	≥16	0.359	0.155	0.834	0.017
Usual number of work hours per week	≤40	0.698	0.545	0.894	0.004
	41–52 (ref.)	1.000			
	53–64	1.130	0.852	1.499	0.396
	≥65	1.431	1.006	2.034	0.046
Satisfaction with income	Satisfactory (ref.)	1.000			
	Neutral	1.396	1.097	1.777	0.007
	Unsatisfactory	2.009	1.561	2.586	0.000
Chi-square (degrees of freedom)		401.094 (19) ***			
Hosmer-Lemeshow test (degrees of freedom)		10.208 (8)			

* Multivariate analysis via binary logistic regression was applied. *** *p* < 0.001.
